# Intermittent PTH administration improves alveolar bone formation in type 1 diabetic rats with periodontitis

**DOI:** 10.1186/s12967-018-1438-2

**Published:** 2018-03-15

**Authors:** Ji-Hye Kim, Ae Ri Kim, Yun Hui Choi, Aeryun Kim, Yongsung Sohn, Gye-Hyeong Woo, Jeong-Heon Cha, Eun-Jung Bak, Yun-Jung Yoo

**Affiliations:** 1 Department of Dental Hygiene, Jeonju Kijeon College, Jeonju, Republic of Korea; 2 Department of Oral Biology, Yonsei University College of Dentistry, 134 Sinchon dong, Seodaemun-gu, Seoul, 120-752 Republic of Korea; 3 Department of Applied Life Science, The Graduate School, Yonsei University, Seoul, Republic of Korea; 4 BK21 PLUS Project, Yonsei University College of Dentistry, Seoul, Republic of Korea; 5 DONG-A Pharm, Yongin-si, Gyeonggi-Do Republic of Korea; 6 Department of Clinical Laboratory Science, Semyung University, Jecheon, Republic of Korea; 7 Microbiology and Molecular Biology, Key Laboratory of Oral Medicine, Guangzhou Institute of Oral Disease, Stomatology Hospital of Guangzhou Medical University, Guangzhou, China

**Keywords:** PTH, Diabetes, Periodontitis, Alveolar bone formation

## Abstract

**Background:**

Periodontitis is an infectious disease that manifests as alveolar bone loss surrounding the roots of teeth. Diabetes aggravates periodontitis-induced alveolar bone loss via suppression of bone formation. Intermittent parathyroid hormone (PTH) administration displays an anabolic effect on bone. In this study, we investigated the effect of intermittent PTH administration on alveolar bone loss in type 1 diabetic rats with periodontitis.

**Methods:**

Rats were divided into control (C), periodontitis (P), periodontitis treated with PTH (P + PTH), diabetes with periodontitis (DP), and diabetes with periodontitis treated with PTH (DP + PTH) groups. To induce type 1 diabetes, rats were injected with streptozotocin and periodontitis was induced bilaterally by applying ligatures to the mandibular first molars for 30 days. During the experimental period, the P + PTH and DP + PTH groups were subcutaneously injected with PTH (40 μg/kg) three times per week, whereas the C, P, and DP groups were injected with citrate buffer. To observe the mineralization of the alveolar bone, the DP and DP + PTH groups were injected with calcein on days 10 and 27, and with alizarin red on day 20. Thirty days after ligation, histological findings and fluorescence labeling were analyzed in the furcations of the mandibular first molars. Sclerostin-positive osteocytes were assessed by immunohistochemical analyses.

**Results:**

The DP groups had smaller areas of alveolar bone than the other groups, and the DP + PTH group had a larger alveolar bone area than the DP group. The DP group had less osteoid formation than the C group, whereas the DP + PTH had greater osteoid formation than the DP group. Fluorescence labeling results revealed that the DP + PTH group had more mineral deposition on the alveolar bone than the DP group. The DP + PTH group exhibited lower percentage of sclerostin-positive osteocytes in alveolar bone than the DP group.

**Conclusions:**

Intermittent PTH administration diminishes alveolar bone loss and sclerostin expression in osteocytes, but increases osteoid formation and mineralization, suggesting that intermittent PTH administration attenuates diabetes-aggravated alveolar bone loss by the induction of bone formation. PTH-induced bone formation may be related to the regulation of osteocytic sclerostin expression in type 1 diabetic rats with periodontitis.

## Background

Parathyroid hormone (PTH), which is synthesized by the parathyroid glands, plays a role in the maintenance of blood calcium levels [1]. Preosteoblasts, osteoblasts, bone-lining cells, and osteocytes express PTH receptors [1]. PTH controls calcium homeostasis via the stimulation of bone remodeling: This process is accomplished by direct effects on osteocytes and osteoblasts, and indirect effects on osteoclasts via its actions on osteocytes and osteoblasts [1]. Although PTH induces both bone resorption and bone formation, its effects are dependent on the frequency of PTH exposure [1]. Continuous and intermittent PTH administration causes catabolic and anabolic effects on bone, respectively [1–3]. PTH-mediated anabolic effects are related to the direct action of PTH on osteoblasts and osteocytes. In osteoblasts, intermittent PTH administration promotes osteoblastogenesis, reduces osteoblast apoptosis, and reactivates quiescent bone-lining cells [1, 4, 5]. In an osteocyte cell line, PTH was shown to reduce sclerostin expression [6]. Sclerostin is mainly expressed by osteocytes, inhibiting Wnt binding to low-density lipoprotein receptor-related protein 5/6 and subsequently decreasing bone formation [7–9]. The suppression of sclerostin expression induced by intermittent PTH administration has been demonstrated in the calvaria of the mice [10].

Periodontitis is an infectious disease induced by periodontopathogens; it causes the loss of alveolar bone surrounding the roots of teeth and is a common cause of tooth loss [11]. Alveolar bone loss is induced by an acceleration of bone resorption and an attenuation of bone formation [11]. Placing ligatures around teeth induces periodontitis in rats, modeling alveolar bone loss [12–14]. Systemic intermittent PTH administration during the maintenance of ligature placement was shown to block alveolar bone loss [12]. The topical intermittent application of PTH in gingiva after the removal of ligatures was shown to recover alveolar bone loss and increase osteoid formation on the alveolar bone surface [13]. These findings suggest an anabolic effect of intermittent PTH administration on alveolar bone in rats with periodontitis.

Systemic diseases such as osteoporosis and diabetes are risk factors for periodontitis [15, 16]. In ovariectomized rats with periodontitis, intermittent PTH administration was shown to reduce alveolar bone loss [17]. PTH-mediated anabolic effects on bone were observed in tibiae and alveolar bone of type 1 diabetic animals [18, 19]. However, the effects of PTH on alveolar bone formation and osteocytic sclerostin expression of rats with both periodontitis and type 1 diabetes have been not determined. Therefore, we investigated the anabolic effects of intermittent PTH administration in rats with streptozotocin (STZ)-induced type 1 diabetes accompanied by periodontitis.

## Methods

### Animal experiments

**Fig. 1:**
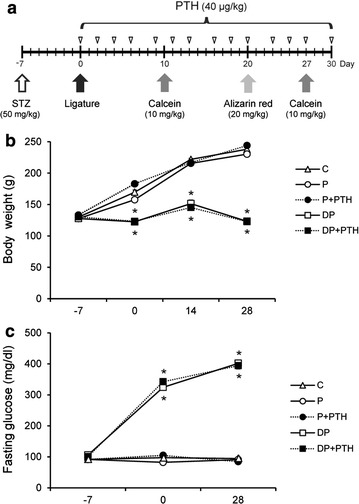
Experimental protocol for intermittent PTH administration and changes in body weights and fasting glucose levels during the experimental period. Rats were divided into five groups: C, P, P + PTH, DP, and DP + PTH. **a** Experimental protocol: on 1 week after induction of diabetes by STZ, periodontitis was induced by placing bilateral ligatures around the mandibular first molars using dental floss. After placement of the ligatures, the DP and DP + PTH groups were given intraperitoneal injections of calcein (10 mg/kg) on days 10 and 27, and alizarin red (20 mg/kg) on day 20, respectively. During 30 days after the induction of periodontitis, the P + PTH and DP + PTH groups were subcutaneously administered PTH three times per week (40 μg/kg). On days 0 and 30, PTH was administered 4 h before both placement of the ligatures and the time of sacrifice, respectively. **b** Body weights in each group and **c** Fasting glucose levels in each group. Data are presented as the mean ± SE. *P < 0.05 compared with the C group

After acclimation for 1 week, the F344 male rats (Japan SLC, Inc., Hamamatsu, Shizuoka, Japan) were divided into five groups: control (C, N = 10), periodontitis (P, N = 7), periodontitis treated with PTH (P + PTH, N = 7), diabetes with periodontitis (DP, N = 6), and diabetes with periodontitis treated with PTH (DP + PTH, N = 6). The experimental protocol is presented in Fig. 1a. After fasting for 18 h, both diabetes groups were intravenously injected with STZ (50 mg/kg in 0.1 M citrate buffer, Sigma-Aldrich, St. Louis, MO, USA) and the non-diabetes groups were injected with citrate buffer alone. On day 7 after injection, blood was obtained from the tail vein, and glucose levels were measured using an Accu-Check active system (Roche, Mannheim, Germany) under fasting conditions. Rats with a blood glucose level greater than 300 mg/dl were considered diabetes. Rats were then anesthetized with zoletil 50 (30 mg/kg; Virbac, Carros, France) and rompun (10 mg/kg; Bayer Korea, Ansan, Gyenoggi-do, Korea), and periodontitis was induced bilaterally by placing dental floss around the cervixes of the first mandibular molars. During 30 days after the induction of periodontitis, the P + PTH and DP + PTH groups were subcutaneously given PTH three times per week (40 μg/kg, Bachem, Torrance, CA, USA). PTH was administered 4 h before both the placement of the ligatures (day 0) and the time of sacrifice (day 30). During the experimental period, body weights and fasting glucose levels were measured to ensure maintenance of diabetes. The animal protocols were approved by the Institutional Animal Care and Use Committee of Yonsei University (Approval Number: 2015-0345).

### Histological analysis of bone loss and bone formation

For histological analysis, the left mandibles and tibiae were fixed in 10% neutral-buffered formalin overnight. The mandibles were decalcified in 10% ethylenediaminetetraacetic acid (EDTA) for 2 months. Serial sagittal sections (4-μm-thick) were cut from paraffin-embedded tissue blocks. The sections with clear dental pulp of the mesial and distal roots of the first molars were selected and stained with hematoxylin and eosin (H&E). Slides were scanned using a Aperio AT2 Digital Whole Slide scanner (Leica Microsystems Inc., Buffalo Grove, IL, USA) and analyzed using Aperio ImageScope software (version12.3.2.2013, Aperio Technologies Inc., Vista, CA, USA). The degree of alveolar bone loss in the furcation of the first molar was estimated by measuring the percentage of the alveolar bone to the region of interest (ROI) in the furcation. The ROI for alveolar bone loss extended 0.8 mm from the top of the furcation. To determine bone formation, the osteoid region of the ROI in the furcation was measured, as previously described [20]. The ROI for bone formation extended 0.5 mm from the remaining alveolar bone crest (ABC) in the furcation. The tibiae of the DP and DP + PTH groups were decalcified in 10% EDTA for 1 month. Tibial sections appearing proximal epiphysial growth plate were selected and then stained with H&E.

### Fluorescence calcein and alizarin red labeling

To analyze bone mineralization, calcein and alizarin red fluorochromes, which bind calcium in the mineralized areas of the bone, were injected into the DP and DP + PTH groups [21]. After placement of the ligatures around the teeth as described above, the DP and DP + PTH groups were given an intraperitoneal injection of calcein (10 mg/kg, Sigma-Aldrich, St Louis, MO, USA) on days 10 and 27, and alizarin red (20 mg/kg, Sigma-Aldrich, St Louis, MO, USA) on day 20, respectively. On day 30 after placement of the ligatures, the right mandibles and tibiae were fixed in 10% neutral-buffered formalin and dehydrated in a graded series of ethanol solutions. The molar regions of the mandibles were embedded in methyl methacrylate-based resin and cut into 200-μm-thick sections (Struers, Germany). The sections were polished to obtain 50-μm slices using a hard tissue grinding system (EXAKT, Germany). Calcein and alizarin red labeling was observed in alveolar bone within the furcation of the first molars and trabecular bone under the epiphyseal growth plate of the tibiae by confocal laser scanning microscopy (LSM 700, Zeiss, Göttingen, Germany).

### Immunohistochemical analyses of sclerostin expression

For sclerostin staining, sections of alveolar bone were deparaffinized and rehydrated through a series of graded ethanol solutions. To inhibit endogenous peroxidase activity, sections were quenched in 3% H_2_O_2_ for 20 min and then treated with trypsin (Thermo Fisher Scientific, Waltham, MA, USA) for antigen retrieval. Immunohistochemistry was carried out using universal kits (Vector Laboratories, Burlingame, CA, USA) according to the manufacturer’s instructions. Sections were preincubated with normal horse blocking solution for 20 min and then incubated overnight at 4 °C with goat anti-sclerostin antibody (1:300 dilution, R&D Systems, Minneapolis, MN, USA). Sections were developed using the 3,3′-diaminobenzidine chromogen and counterstained with methyl green. Slides were scanned using a Aperio AT2 Digital Whole Slide scanner (Leica Microsystems Inc., Buffalo Grove, IL, USA) and analyzed using Aperio ImageScope software (version12.3.2.2013, Aperio Technologies Inc., Vista, CA, USA). Numbers of sclerostin-positive osteocytes were counted in the ROI, which extended 0.5 mm from the ABC in the furcations of the first molars, and are presented as the percentage of sclerostin-positive osteocytes per number of total osteocytes in the ROI.

### Statistical analysis

All statistical analyses were performed using a statistical analysis program (SPSS, Chicago, IL, USA). One-way analysis of variance followed by Scheffé’s method was used to determine significant differences. A value *P* < 0.05 was considered statistically significant. Data are expressed as the mean ± standard error (SE).

## Results

### Effects of PTH on body weights and fasting blood glucose levels

During the experimental period, C, P, and P + PTH groups showed a gradual increase in body weights, but the DP and DP + PTH groups maintained body weights (Fig. 1b). Fasting blood glucose levels of the C, P, and P + PTH groups were about 100 mg/dl and were maintained during the experimental period (Fig. 1c). However, fasting blood glucose levels of the DP and DP + PTH groups were greater than 300 mg/dl. There was no difference in body weights and fasting glucose levels between the DP and DP + PTH groups.

### Effects of PTH on alveolar bone loss and bone formation

**Fig. 2:**
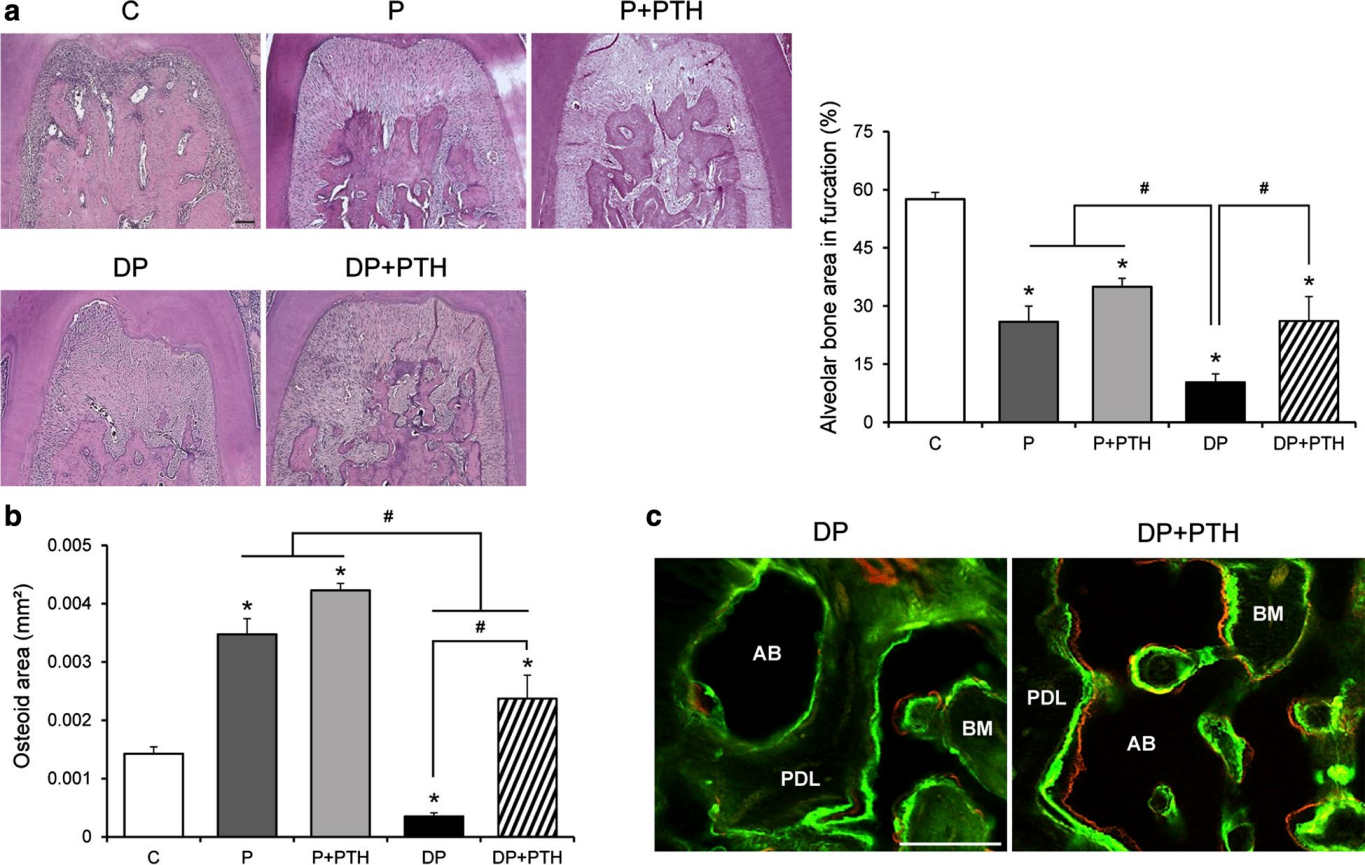
The effects of intermittent PTH administration on alveolar bone loss and bone formation in the furcations. **a** Representative images of the alveolar bone area in each group (left panels; H&E stain; scale bar = 100 μm). Measurements of the alveolar bone area in each group (right panel). **b** Measurements of the osteoid area in each group. **c** Representative images of labeled alveolar bone surfaces by calcein and alizarin red fluorescence in the DP and DP + PTH groups (scale bar = 100 μm). Data are presented as the mean ± SE. *P < 0.05 compared with the C group. ^#^*P* < 0.05. *AB* alveolar bone; *BM* bone marrow, *PDL* periodontal ligament

To estimate the effects of PTH on alveolar bone loss, the alveolar bone areas in the furcations of the first molars were measured (Fig. 2a). The alveolar bone areas of the P, P + PTH, DP, and DP + PTH groups were lower than the corresponding area in the C group. Interestingly, the DP + PTH group had more alveolar bone than did the DP group. To evaluate the effects of PTH on new bone formation in alveolar bone, the osteoid region was estimated in the furcations of the first molars (Fig. 2b). While the osteoid area was larger in the P, P + PTH, and DP + PTH groups than in the C group, it was smaller in the DP group. Both the P and P + PTH groups had the larger osteoid areas than did the DP and DP + PTH groups. The DP + PTH group had the greater osteoid area than the DP group. Calcein- and alizarin red-labeled bone areas were larger in the DP + PTH group than in the DP group (Fig. 2c).

### Effects of PTH on tibial bone loss and bone formation

**Fig. 3:**
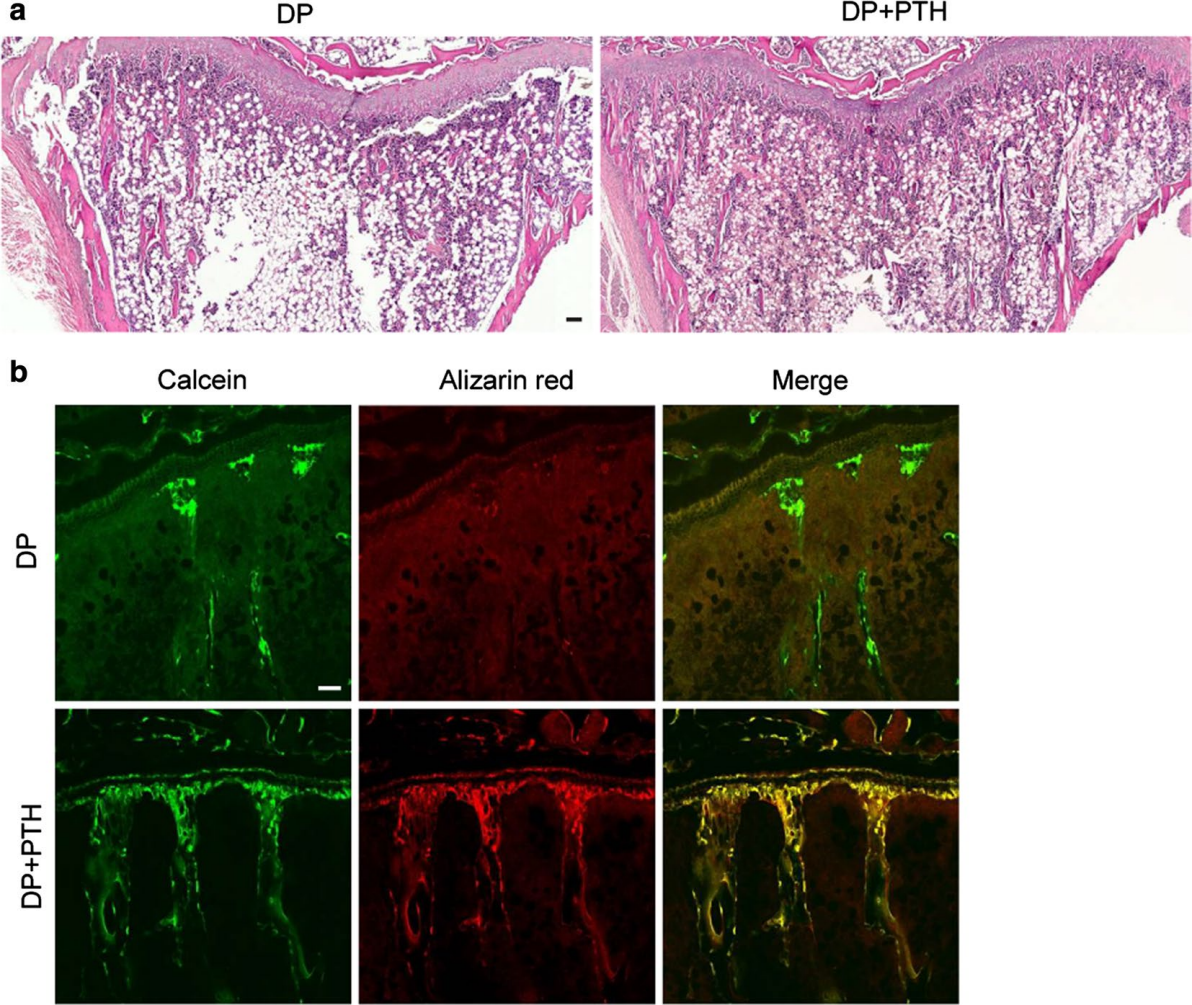
The effects of intermittent PTH administration in tibiae. **a** Representative images of trabecular bone in tibiae of the DP and DP + PTH groups (H&E stain; scale bar = 100 μm). **b** Representative images of labeled trabecular bone surfaces by calcein and alizarin red fluorescence in the DP and DP + PTH groups (scale bar = 100 μm)

To confirm the anabolic effects of PTH on other bone, trabecular bone in the tibiae was observed by H&E staining and fluorescence labeling. The DP + PTH group showed more trabecular bone than the DP group by H&E staining (Fig. 3a), and the DP + PTH group showed a larger calcein- and alizarin red-labeled area in trabecular bone than the DP group (Fig. 3b).

### Effects of PTH on sclerostin expression in alveolar bone

**Fig. 4:**
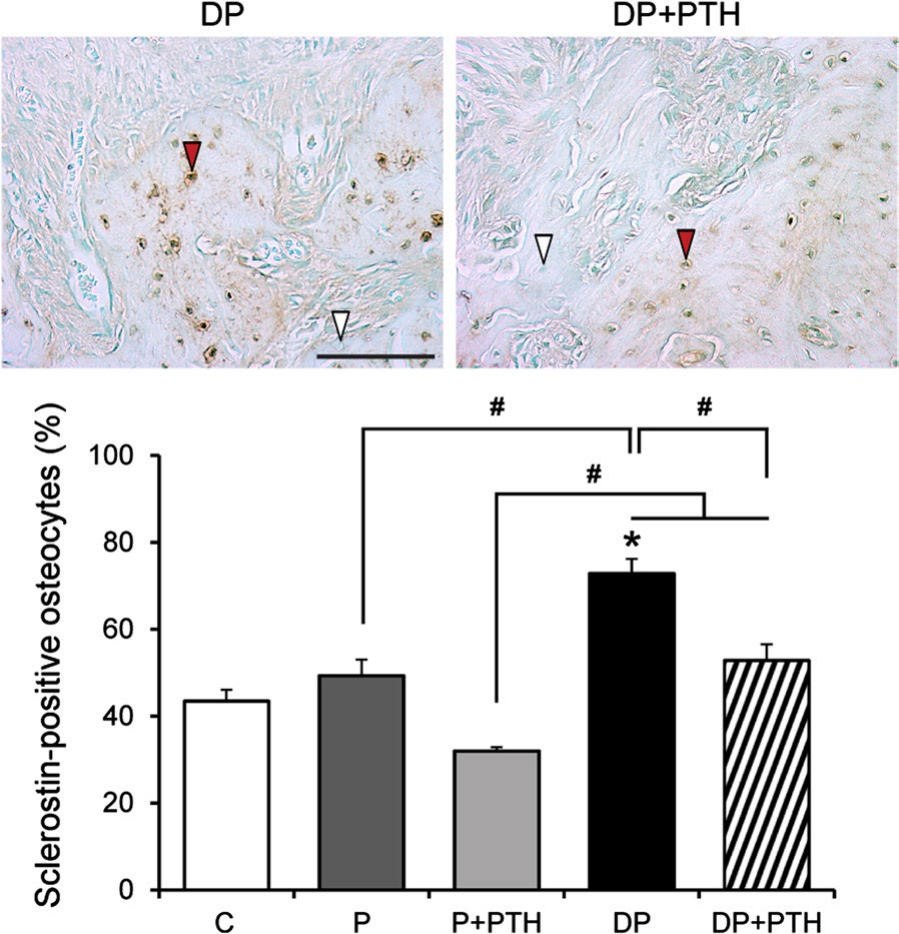
The effects of intermittent PTH administration on osteocytic sclerostin expression in alveolar bone. Representative images of sclerostin-positive osteocytes in the DP and DP + PTH groups. White and red arrowheads indicate representative sclerostin-negative and -positive osteocytes, respectively (upper panels; immunohistochemistry; scale bar = 100 μm). Percentage of sclerostin-positive osteocytes in each group (lower panel). Data are presented as the mean ± SE. *P < 0.05 compared with the C group. ^#^*P* < 0.05

To estimate the effects of PTH on sclerostin expression in alveolar bone, sclerostin-positive osteocytes were counted in the furcations of the first molars (Fig. 4). The percentage of sclerostin-positive osteocytes was greater in the DP group than in the other groups. While that of DP + PTH group was lower than in the DP group, it was higher than in the P + PTH group.

## Discussion

The frequency of PTH administration required to elevate PTH levels intermittently varies from daily to a few times per week. Daily PTH administration increases osteoblast number and bone formation in rat tibiae, whereas continuous PTH infusion via an osmotic pump results in systemic effects, including bone resorption and bone marrow fibrosis, suggesting that intermittent PTH administration is required for the anabolic effects of this hormone [2]. In both rats with periodontitis and ovariectomized rats with periodontitis, anabolic effects on alveolar bone were induced by PTH administration three times per week for 1 month [12, 17]. Based on the data generated by these two groups, we administered PTH three times per week for 1 month in type 1 diabetic rats with periodontitis. PTH administration in periodontitis condition did not result in a significant increase in the area of the alveolar bone compared to the P group, but PTH administration in periodontitis with diabetes condition elicited its improvement compared to the DP group. These findings suggest that intermittent PTH administration may attenuate diabetes-aggravated alveolar bone loss in type 1 diabetic rats with periodontitis. A study of PTH-treated ovariectomized rats with periodontitis (three times per week) showed recovery of alveolar bone loss to levels of normal rats [17]; these results are inconsistent to our findings, where alveolar bone loss in the DP + PTH group did not recover to levels of the C group. The characteristics of systemic diseases related to bone loss might contribute to differences in the level of protection against alveolar bone loss. Additionally, daily PTH administration in mice with type 1 diabetes caused an elevation of trabecular bone density in the tibiae to levels of normal mice [18]. To improve rates of bone protection in cases of type 1 diabetes with periodontitis after PTH administration, the optimal therapeutic strategy considering bone type in PTH administration might be considered.

Bone loss was determined by the level of bone formation. As in study of rats with periodontitis treated intermittently with PTH [13], we also found greater osteoid area in alveolar bone of the DP + PTH group compared to the DP group. In addition, mineral deposition in alveolar bone was greater in the DP + PTH group than in the DP group, suggesting an anabolic effect of PTH on alveolar bone in type 1 diabetic rats with periodontitis. In long bones of type 1 diabetic mice, daily PTH administration increased trabecular bone density accompanied by increased mineral apposition and an increase in the number of osteoblasts lining the bone surface [18]. In our study, tibiae of the DP + PTH group had an increase in trabecular bone and mineral deposition compared with the DP group. These findings suggest that PTH has similar anabolic effects on trabecular bone (e.g., the tibia) as on alveolar bone in type 1 diabetes.

In previous studies of rats with periodontitis, treatment with sclerostin antibodies was found to induce an anabolic response during alveolar bone healing by increasing bone formation [15, 22]. Sclerostin in osteocytes is proposed to be a target gene of PTH in vivo [1, 6, 10]. Daily PTH administration has been shown to increase osteoblast activity in normal mice, with no effects in mice lacking PTH receptors in osteocytes [23]. In addition, a single PTH administration was shown to decrease the number of sclerostin-positive osteocytes in vertebral trabecular bone in normal mice but failed to suppress the number of sclerostin-positive osteocytes in mice lacking PTH receptors [23]. These results suggest that osteocytes play a significant role in the anabolic effects of intermittent PTH administration. A single PTH administration was found to induce a transient decline in sclerostin mRNA levels in mice vertebrae and rat femurs [6, 10]. Daily PTH administration for 2 months was found to induce a steady decline in sclerostin mRNA levels in femurs of estrogen-deprived rats, suggesting that inhibition of sclerostin is sustained by long-term intermittent PTH administration [10]. In this study, more sclerostin-positive osteocytes were observed in the DP group compared to the C group. There was no significant difference in sclerostin expression in the P + PTH group compared to the P group, but sclerostin expression was lower in the DP + PTH group compared to the DP group. Our results suggest that PTH may have an inhibitory effect on the induction of sclerostin expression in alveolar bone by diabetes similar to results seen in a study of estrogen-deprived rats treated with PTH [10]. Taken together, decreased sclerostin expression in osteocytes after intermittent PTH administration appears to be involved in the increase in bone formation observed in type 1 diabetic rats with periodontitis.

## Conclusions

In summary, these results indicate that intermittent PTH administration increases osteoid formation and mineralization, and diminishes alveolar bone loss and sclerostin expression in osteocytes of type 1 diabetic rats with periodontitis. Therefore, intermittent PTH administration may improve bone formation and attenuate diabetes-aggravated bone loss by decreasing sclerostin expression in osteocytes of type 1 diabetic rats with periodontitis.

## Abbreviations

ABC: alveolar bone crest
C: control
DP: diabetes with periodontitis
DP + PTH: diabetes with periodontitis treated with PTH
EDTA: ethylenediaminetetraacetic acid
H&E: hematoxylin and eosin
N: number
P: periodontitis
P + PTH: periodontitis treated with PTH
PTH: parathyroid hormone
ROI: the region of interest
SE: standard error
STZ: streptozotocin
